# Patient-level factors influencing hypertension control in adults in Accra, Ghana

**DOI:** 10.1186/s12872-020-01370-y

**Published:** 2020-03-11

**Authors:** Darlene Esinam Okai, Adom Manu, Emefa Modey Amoah, Amos Laar, Joseph Akamah, Kwasi Torpey

**Affiliations:** 1 University of Ghana School of Public Health, Accra, Ghana; 2 University of Ghana School of Medicine and Dentistry, Accra, Ghana

**Keywords:** Hypertension, Blood pressure control, Pill burden, Ghana, Developing countries

## Abstract

**Background:**

Effective control of blood pressure is necessary to avert the risk of cardiovascular diseases from uncontrolled hypertension. Despite evidence on the benefits of hypertension control, rates of control in Ghana remain low. This study assessed the patient-level factors that influence hypertension control among adults in Accra, Ghana.

**Methods:**

A total of 360 hypertensive patients from two hospitals in Accra, Ghana were enrolled in the study. Patient socio-demographic characteristics were tabulated and associations between patient characteristics and hypertension control were estimated using chi-square tests and logistic regression.

**Results:**

Less than a quarter of the patients had a controlled blood pressure. The patient’s sex [AOR = 3.53 (95% CI:1.73–7.25], educational at junior high school [AOR = 3.52(95% CI 1.72–7.22)], senior and junior high school [AOR = 2.64 (95% CI 1.40–6.66_] and AOR = 3.06 (95% CI 1.03–6.67)] and presence of a comorbidity [AOR = 2.41 (95% CI 1.32; 4.42)] predicted BP control among patients. Dyslipidaemia [AOR = 0.31, [0.11–0.89)] an increased pill burden, and length of diagnosis of 2–5 years (AOR = 0.27 (0.1–0.73)] however, were associated with reduced BP control [AOR = 0.32(95% CI: 0.18–0.57)]. The majority of patients reported forgetfulness, side effects of medication and high pill burden as reasons for missing their medications.

**Conclusion:**

Knowledge of hypertension among patients is low. Sex, formal education and the presence of comorbidity and more specifically dyslipidaemia influences blood pressure control. High pill burden and 2–5 years since diagnosis negatively affects the attainment of blood pressure control.

## Background

Hypertension is recognized as a public health issue worldwide. Despite the global policy agenda set to address this challenge, blood pressure control remains elusive and hypertension is the leading cause of cardiovascular disease (CVD) worldwide [1–4]. Although there is evidence to show that awareness, treatment, and adherence to antihypertensive medication are essential for hypertension control [5], all three factors remain low across different countries and various settings [6]. Africa, compared to the rest of the world, has the highest hypertension prevalence with 27% of adults being hypertensive [7].

Unlike more developed countries of the world, low and middle-income countries (LMICS) continue to experience significant mortalities from infectious diseases [8]. The epidemiologic transition in developing countries leaves in its wake a myriad of chronic diseases. The occurrence of CVDs in the presence of prevalent infectious diseases contributes to increased mortality and places many developing countries into a double burden of disease [9].

In Ghana, the Ghana Health Service (GHS) 2014 Annual Report attributed the highest outpatient cases between 2011 and 2014 to hypertension. Within that same period, the Greater Accra and Ashanti region recorded the highest number of diagnosed cases of hypertension totalling 152,545 and 140,947 respectively. In Ghana, 63% of women and 86% of men that are hypertensive reported being unaware of their condition according to the Ghana Demographic Health Survey 2014 [10]. As a preventive measure, the number and proportion of facilities running NCD Clinics and providing screening services for hypertension and diabetes were increased.

Despite the existence of extensive evidence on the benefits of hypertension treatment, control has been unsatisfactory [1, 11, 12]. Worldwide, assessments show less than 50% of treated hypertensive patients reach their blood pressure goals; increasing their risk of hypertension-induced complications [13]. Recent studies on hypertension control have shown that patient, clinician as well as the health environment/health system factors all influence the attainment of hypertension control [14–16].

Control rates of hypertension vary widely across and within populations. Various studies have reported rates of 32.9–49% among similar population groups [1, 16–18]. In Ghana, however, control rates reported are relatively lower and range from 24 to 42% [19–21]. The available studies that have established the high prevalence of uncontrolled hypertension in Ghana but found inconsistent associations between socio-demographic factors and blood pressure control [20, 21]. Where patient level factors have been assessed, evidence shows poor control associated with the number of hypertensive medications, duration of diagnosis and access to medication [21].

The Seventh Report of the Joint National Committee (JNC 7) recommends that all patients be classified as normal (120/< 80), pre-hypertensive (120–80 – 139/89), or hypertensive based on the level of the systolic and/or diastolic Blood Pressure (BP). Hypertension control is therefore achieved when a hypertensive individual attains blood pressure targets of less than 140/90 mmHg. For patients with co-morbidities such as diabetes and/or CKD, targets are set even lower at < 130/80 mmHg for beneficial treatment outcomes [22].

Target 6 of the nine endorsed global NCDS targets, call for member states to achieve a 25% reduction in elevated blood pressure within their specific settings [4]. Reducing hypertension-related mortality, therefore, requires that the proportion of patients with controlled hypertension increases. This study, therefore, focuses on patient-level factors influencing blood pressure control among hypertensive adults in the Greater Accra Region of Ghana. Data from this study will support calls to develop comprehensive policies that will inch the country closer to achieving the global targets of control of the condition and reducing the resulting mortalities.

## Methods

### Study design

This was a cross-sectional survey of adult hypertensive patients attending two hospitals in the Greater Accra Region of Ghana.

### Participants

A total of 360 participants were interviewed in this study – 213 from the first and 147 from the second hospital respectively. Only adult subjects were included in the study (18 years and above).

Inclusion criteria: Hypertensive patients who have been on treatment for not less than a year.

Exclusion criteria: Pregnant women, patients who were taking drugs that could increase blood pressure and patients diagnosed with psychiatric conditions were excluded from the study.

### Sampling procedure

A list of hypertensive patients given appointments for each day was obtained from the records department of both facilities. Numbers were assigned to the individuals on the list. The numbers were randomly selected using the Excel random number generation. An average of 50 to 60 patients was booked for each clinic day for both facilities with the facility with a higher caseload contributing 30–35 per day.

### Data collection method/ technique and tools

A written informed consent (signed or thumb printed) was obtained from each participant Respondents who were unable to read, write or sign nominated an independent witness to attest to the consent process. Data was collected using a structured questionnaire composed of four different sections (Additional file 1). The first section of the questionnaire recorded a minimum of two and a maximum of five BP readings inclusive of previous visits to the hospital. This section was completed after accessing this information from the individual patient folders. The BP readings recorded were taken by the healthcare provider as part of the routine care provided at the clinic. Blood pressure readings were taken in the non-dominant arm using an automated sphygmomanometer with the patient in an upright sitting position after having rested for at least 10 min. Blood pressure classification for patients with more than one visit was based on the averaging of 2–5 recorded readings [22, 23].

The second section consisted of questions on the socio-demographics of participants including age, sex, marital status, level of education and income level. Section three examined patients’ knowledge about hypertension, its causes, and complications as well as comorbidities (dyslipidaemia, diabetes, stroke, and chronic kidney disease) and the associated medications. Information about the taking of non-prescribed drugs was also obtained. A final knowledge score was obtained for each respondent out of 13. The categorisation of knowledge was based on a tercile distribution. The respondents were scored on their knowledge of a normal adult blood pressure and the number of causes and complications of hypertension they knew of. Scores of 9–13 (representing the upper third) were considered as having “Good knowledge”, scores of 5–8 (representing the middle third) were categorized as “Moderate knowledge” and scores of 1–4 (representing the lower third) were categorized as “Poor knowledge”.

The next section assessed patients’ perceptions about the control status of their hypertension as well as their perception of the efficacy of antihypertensive medications being taken. The final section sought to assess patient level factors such as adverse effects experienced from the use of antihypertensive medication, high pill burden /polypharmacy, cost of medications, forgetfulness, and belief in divine intervention or belief in being cured of hypertension.

The developed questionnaire was reviewed by subject matter experts for face and content validity for readability, clarity and comprehensiveness. Minor revisions were necessary. The tool was further revised, and pretested among hypertensive patients reporting for blood pressure management at a facility that served a similar population to the study areas. This was to determine performance in the field as planned [24].

### Data analysis

Cross-tabulations of patient socio-demographic variables, factors influencing hypertension control were undertaken. A descriptive analysis was conducted using Pearson’s χ^2^ and fishers exact where appropriate. The hypertension control status of each patient was determined by calculating the average of all SBP and DBP readings recorded. Binary logistic regression was conducted on socio-demographic and patient level factors to determine their influence on hypertension control. All predictor variables were entered in the regression model at once. Blood pressure control in logistic regression was coded as 0 for poor control and 1 for optimal control. All analyses were conducted in STATA and statistical significance was set at a 95% confidence interval.

### Ethical considerations

Ethical clearance was sought from the Ghana Health Service Ethical Review Committee with approval number (GHS-ERC: 037/12/17). Permission to conduct research at Ghana Health Service facilities was also sought from the Regional Directorate of Ghana Health Service, Greater Accra region and subsequently from the Municipal Directorates of both facilities.

## Results

### Socio-demographic characteristics of respondents

A total of 360 patients participated in the study. All participants resided in urban areas and were native Ghanaians. By sex, 105 males and 255 females were interviewed with the mean age of 61.9 (± 10.7) with ages ranging from 27 to 94 years. More than half of the respondents were aged over 61 years. Respondents were mostly married, and few had attained tertiary education (4.7%) (Table 1).

**Table 1:** Socio-demographic characteristics of respondents

Characteristic	Frequency (n = 360)	Percentage (%)
Age		
Mean age (± SD)	61.9 ± 0.56	
27–45	19	5.28
46–60	142	39.44
61–94	199	55.28
*Sex*		
Male	105	29.17
Female	255	70.83
Knowledge		
Poor knowledge	264	73.33
Moderate knowledge	92	25.56
Good knowledge	4	1.11
Marital Status		
Single	20	5.56
Married	243	67.50
Divorced	20	5.56
Widowed	77	21.39
Educational Status^a^		
No formal education	92	25.56
Primary	109	30.28
Junior high	85	23.61
Secondary	57	15.83
Tertiary	17	4.72
Income (US $)		
Less than $40	209	58.06
$40- $120	112	31.11
$120–$210	30	8.33
> $210	9	2.50

^a^Primary: the first 6 years of formal education as primary. Junior high: year 7–9, Secondary: years 10–12. Tertiary: higher education

### Respondents’ knowledge about hypertension

Approximately 73.3% of respondents showed poor knowledge of hypertension, 25.6% showed moderate knowledge and only 1.1% showed good knowledge of hypertension (Table 1).

### Reasons for missing medication among respondents

Table 2 presents the reported reasons for missed medication among the respondents. The majority (70.5%) cited forgetfulness as the reason for missing their medication. Another reason that was prominently cited was the high pill burden.

**Table 2:** Reasons for missed medication among respondents (*n* = 291^a^)

Reason	Frequency	Percentage
Forgetfulness	205	70.45
High pill burden	78	26.80
Side effects of medication	69	23.71
Cost of medication	12	4.12
Does not believe in orthodox medicines	16	5.50
Believed was cured	9	3.09
Believe in divine intervention	1	0.34

^a^ Available for 291 participants who responded to reason for missing medication. Multiple response permitted

### Hypertension control among respondents

Less than a quarter of patients achieved hypertension control (23.3%). Various socio-demographic characteristics were assessed against hypertension control. By sex, among female respondents, 69 (27.1%) had achieved hypertension control and 15 (14.3%) of male respondents had achieved hypertension control. This association was found to be significant (*p* < 0.009). Table 3 contains further details on hypertension control across various socio-demographic factors.

**Table 3:** Socio-demographic factors and Hypertension control

Variable	Controlled BP			
	No	Yes	χ2	*p*-value
	n (%)	n (%)		
Age				
Mean age (± SD)	61.2 ± 0.61	64.1 ± 1.31		0.012
Age categories				
27–45	15(78.95)	4(21.05)	0.7991	0.671
46–60	112(78.87)	30(21.13)		
61–94	149(74.87)	50(25.13)		
Sex				
Male	90(85.71)	15(14.29)	6.7832	0.009
Female	186(72.94)	69(27.06)		
Marital Status				
Single	15(75.00)	5(25.00)	5.9367	0.115
Married	195(80.25)	48(19.75)		
Divorced	13(65.00)	7(35.00)		
Widowed	53(68.83)	24(31.17)		
Knowledge				
Poor knowledge	205(77.65)	59(22.35)		0.701^b^
Moderate knowledge	68(73.91)	24(26.09)		
Good knowledge	3(75.0)	1(25.0)		
Educational Status^a^				
No formal education	74(80.43)	18(19.57)	8.4273	0.077
Primary	90(82.57)	19(17.43)		
Junior high	56(65.88)	29(34.12)		
Secondary	43(75.44)	14(24.56)		
Tertiary	13(76.47)	4(23.53)		
Income				
Less than $40	163(77.99)	46(22.01)	0.95	0.781
$40- $120	84(75.00)	28(25.00)		
> $120	29 (74.36)	10(25.64)		
Total	276 (76.67)	84 (23.33)		

n: Frequencies, %:row percentages

^a^Primary: the first 6 years of formal education as primary. Junior high: year 7–9, Secondary: years 10–12. Tertiary: higher education

^b^ Based on Fishers exact test

### Associations between patient factors and hypertension control

Assessing patients by comorbidity showed that 18% of patients who had no comorbidities had achieved hypertension control. However, more patients with some comorbidity had achieved hypertension control (23.3%). Among patients with dyslipidaemia, 8.9% had controlled hypertension (*p* < 0.006). Table 4 has further details of factors associated with hypertension control.

**Table 4:** Associations between other patient factors and hypertension control

Variable	Controlled BP			
	No	Yes	χ2	*p*-value
	n(%)	n(%)		
Comorbidity				
No	59(62.11)	36(37.89)	15.2969	< 0.001
Yes	217(81.89)	48(18.11)		
Diabetes				
No	147(74.62)	50(25.38)	1.0195	0.313
Yes	129(79.14)	34(20.86)		
CKD				
No	262(75.72)	84(24.28)		0.047^c^
Yes	14(100.00)	0(0.00)		
Dyslipidaemia				
No	225(74.01)	79(25.99)	7.6921	0.006
Yes	51(91.07)	5(8.93)		
Stroke				
No	262(76.83)	79(23.17)	0.0997	0.752
Yes	14(73.68)	5(26.32)		
Pills taken for comorbidity				
1–2	84(80.77)	20(19.23)		0.725^c^
3–6	130(83.87)	25(16.13)		
7–13	3(75.00)	1(25.00)		
Non-Prescribed Med^b^.				
No	209(76.56)	64(23.44)	0.0076	0.930
Yes	67(77.67)	20(22.99)		
Length of diagnosis				
1	21(63.64)	12(36.36)	11.9293	0.008
2–5	112(84.21)	21(15.36)		
6–10	91(78.45)	25(21.55)		
21–40	52(66.67)	26(33.33)		
No. BP pills taken per day				
1–2	85(64.89)	46(35.11)	15.978	< 0.001
2–4	191(83.41)	38(16.59)		
Total	276 (76.67)	84 (23.33)		

n: Frequencies,%:row percentages

^**a**^ Co-morbidity was defined as the presence of diabetes, chronic kidney disease or dyslipidaemia

^b^Non-prescribed medication refers to any drug the patient is currently taking that is not prescribed by the attending physician

^c^ Fishers’ exact value

### Determinants of hypertension control

Table 5 shows the determinants of hypertension control among patients with their crude and adjusted odds ratios (AOR). Females were 3.55 times more likely to have their BPs controlled compared to men [AOR = 3.55 (95% CI 1.72, 7.22)]. A 69% reduction in the odds of having controlled hypertension was identified among patients who suffered from dyslipidaemia as comorbidity, compared to those who did not suffer this comorbidity [AOR = 0.31 (95% CI 0.11, 0.89)].

**Table 5:** Socio-demographic determinants of hypertension control among respondents

Characteristic	UOR [95% CI]	AOR ^a^ [95% CI]
Age		
27–45	Ref	Ref
46–60	1.00 [0.31–3.25]	2.18[0.50–9.52]
61–94	1.25[0.40–3.97]	2.04[0.46–9.02]
Knowledge		
Poor knowledge	Ref	Ref
Moderate knowledge	1.22[0.71–2.12]	1.05[0.54–2.06]
Good knowledge	1.56[0.12–11.34]	1.22[0.08–19.54]
Sex		
Male	Ref	Ref
Female	2.23[1.21–4.11]*	3.33[1.65–6.71]**
Education		
No formal Education	Ref	Ref
Primary	0.88[0.42–1.77]	1.24[0.55–2.78]
Junior High	2.13[1.08–4.21]*	3.06[1.4–6.66]*
Secondary	1.34[0.61–2.96]	2.64[1.03–6.76]*
Tertiary	1.27[0.37–4.34]	1.51[0.32–7.09]
Length of diagnosis (years)		
1	Ref	Ref
2–5	0.33[0.14–0.77]*	0.27[0.1–0.73]*
6–10	0.48[0.21–1.11]	0.52[0.19–1.4]
11–40	0.88[0.37–2.05]	0.67[0.23–1.91]
Comorbidity		
Comorbidity present	2.76[1.64–4.64]*	0.41[0.22–0.75]**
Dyslipidaemia		
Dyslipidaemia Absent	Ref	Ref
Dyslipidaemia Present	0.28[0.11–0.72]**	0.31[0.11–0.89]*
Stroke		
Stroke absent	Ref	Ref
Stroke Present	1.18[0.41–3.34]	1.9[0.57–6.38]
No. BP pills taken per day		
1–2 pills	Ref	Ref
3–4 pills	0.37[0.22–0.61]***	0.32[0.18–0.57]**

*UOR* Unadjusted odds ratio ^a^ Adjusted for all items in the table

*AOR*: Adjusted odds ratio* *p* < 0.05; ** *p* < 0.01; ***p < 0.001

*Primary: the first 6 years of formal education as primary. Junior high: year 7–9, Secondary: years 10–12. Tertiary: higher education

Taking a higher number of antihypertensive pills per day was also associated with a reduced likelihood of attaining hypertension control. Given that a respondent took 3–4 antihypertensive pills per day, the odds of having a controlled BP was reduced by 68% [AOR = 0.32 (95% CI 0.18, 0.57)] compared to those who took 1 to 2 pills. (Table 5).

## Discussion

The findings of this study are indicative both of low knowledge of hypertension and blood pressure control among patients. Factors that were significantly associated with BP control were sex, education and the presence of comorbidity. Females achieved BP control more than their male counterparts, a finding in congruence with recent evidence from the Ghanaian setting [19, 20]. This improved control among females could be the result of higher levels of awareness and treatment of hypertension among females than males [19]. It is nonetheless a worrying observation considering the higher incidence of stroke among Ghanaian males than females [25].

Although knowledge of hypertension in this study was critically lower than available estimates, it is inconsistent with reports indicating poor knowledge of hypertension among Ghanaians [20, 21, 26]. These low levels of knowledge are nonetheless comparable to levels recorded in South Africa where only 0.3% of respondents were found to have good knowledge [27]. Surprisingly whereas others have found significant associations between knowledge of hypertension and hypertension control [28, 29], no significant association between knowledge and hypertension control was observed in this study. These findings are similar to other studies conducted in Southern Africa [27, 30]. It is plausible that hypertension control levels among participants were low due to the poor knowledge of hypertension reported. This is particularly notable as good knowledge of hypertension has been reported to improve compliance to antihypertensive treatment and consequently the control of the disease [31].

In this study, blood pressure control was positively associated with junior high and secondary education compared to those with no formal education. A finding in contrast to recent district-level findings in Ghana but indicative of opportunities for health education among patients that have attained at least junior secondary education [21].

A larger proportion of patients in this study with comorbidities did not achieve BP control. It is plausible, that comorbid patients, may have encountered challenges in observing the treatment for blood pressure control. This finding is similar to reported studies that highlight reduced BP control among patients with dyslipidaemia [32, 33]. It is plausible that among such patients, treatment of the comorbidity may be suboptimal. This poses a challenge to the successful control of hypertension among such patients.

Among patients who report taking 3–4 blood pressure pills per day, the observed reduction in control may be a result of the challenges of high pill burden. Similar results from more developed settings showed that patients who were on three or more antihypertensive pills were likely to miss their medication or have a treatment gap compared to those who were being treated on two pills [34]. Although in some settings, patients are educated to appreciate the severity of their condition to mitigate these treatment gaps [35]. Furthermore evidence indicates reduced BP control with an increasing number of antihypertensive medications [21]. Blood pressure Control among patients with high pill burdens could be improved with the use of fixed-dose combination (FDC) poly-pills to simplify treatment [36–38].

The reported reasons for missed medications highlighted forgetfulness as the prime cause. For adult patients, it is plausible that forgetfulness is due to the competing psychosocial demands of daily life. Medication side effects, high pill burden, cost of medication, belief in divine intervention, belief that one is cured and distrust of orthodox medicine were reasons given by respondents for poor adherence. These reasons are nonetheless similar to those identified in a Nigerian study on hypertension medication adherence [18].

Our study is not without limitations. First, information concerning knowledge and management of hypertension was self-reported lending itself to recall and information bias. All other patient history and comorbidity was reviewed from records. This study also does not control for the different treatment types being used by participants on treatment. We recommend that physician-patient communication on the importance of treatment for the control and improvement in the condition must be encouraged to foster awareness and adherence. There is also a need for treatment guidelines with an emphasis on the use of polypills to reduce pill burden and improve blood pressure control.

## Conclusion

This study concludes that knowledge of hypertension among patients is low. Although sex, formal education and the presence of comorbidity and more specifically dyslipidaemia influences blood pressure control. High pill burden negatively affects the attainment of blood pressure control. The study also adds to the growing body of evidence highlighting the consistently low rates of hypertension control in Ghana over the last decade [39].

## Supplementary information

**Additional file 1.** English questionnaire developed for the study.

## Data Availability

The datasets used and analysed during the current study are available from the corresponding author on reasonable request.

## Abbreviations

AOR: Adjusted odds ratio
BP: Blood pressure
CI: Confidence interval
CKD: Chronic kidney disease
CVD: Cardiovascular disease
DBP: Diastolic blood pressure
FDC: Fixed-dose combination
GHS: Ghana health services
LEKMA: Ledzokuku Krowor municipal assembly
LMICS: Low and middle-income countries
NCDs: Non-communicable diseases
SBP: Systolic blood pressure
