# Supplementary material for: Patient-level factors influencing hypertension control in adults in Accra, Ghana
Source: BMC Cardiovasc Disord. 2020 Mar 11;20:123. doi: 10.1186/s12872-020-01370-y (PMC7065309; doi:10.1186/s12872-020-01370-y)
Supplement: Supplementary file 1 — Additional file 1. English questionnaire developed for the study. [file 12872_2020_1370_MOESM1_ESM.docx]

## Questionnaire

**Project Title: Patient-level factors influencing hypertension control in adults in Accra-Ghana.**

| Date |  |
| --- | --- |
| Respondent’s Identification Number |  |
| Interviewer code |  |
| **SECTION 1 : BP READINGS** | |
| **BP Readings** | **Date** |
| 1. |  |
| 2. |  |
| 3. |  |
| 4. |  |
| 5. |  |

| **SECTION 2: SOCIO-DEMOGRAPHICS OF PATIENTS** |
| --- |
| 1. **Sex**   Male [0] Female [1] |
| 1. **What is your age (at last birthday)?** |
| 1. **What is your marital status?**  - Single [1] Married [2] Divorced [3] Widowed [4] |
| 1. **What is your level of education?**   No Formal Education [1] Primary [2] Junior High [3] Secondary [4] Tertiary [5] |
| 1. **What is your occupation?**  - Unemployed [1] Trader / Artisan [2] Professional [3] Retired [4] |
| 1. **How much is your monthly income?**  - Less than GHS 200 - GHS200- 600 - GHS600-1000 - >GHS1000 |
| **7**. **Where do you live?**  **………………………………………………..** |
| 8**. What is your religion?**   - Christian [1] - Muslim [2] - Traditional [3]   Other [4] ………… |

**SECTION 3: KNOWLEDGE ABOUT HYPERTENSION AND TREATMENT**

| 1. **How long ago were you diagnosed as hypertensive** (in years)**?** |  |
| --- | --- |
| 1. **What is a normal adult blood pressure?** | - 120/80 [1] Don’t know / Other[2] |
| *TICK ALL THE ANSWERS THAT APPLY* | |
| 1. **What do you think are the causes of hypertension?** | - Inherited from the Family  [1] - Lack of Exercise [2] - Age [3] - Poor Diet [4] - Alcohol Use [5] - Smoking [6] - Stress [7] |
| 1. **What do you think are the complications of hypertension?** | - Stroke [1] - Heart Failure [2] - Renal Failure [3] - Erectile Dysfunction [4] - Foot Ulcer [5] |
| 1. **Apart from hypertension, do you have any of these medical conditions?** | - None [0] - Diabetes [1] - Heart Disease/Failure [2] - Stroke [3] - Chronic Kidney disease [4] - Asthma [5] - High cholesterol [6] |
| 1. If you answered yes to (Question 5) what are the total number of pills you take for that condition in a day? |  |
| 1. **Do you take non prescribed drugs?** | - Yes [1] - No [2] |

**SECTION 5: PATIENT’S BELIEFS AND MOTIVATION**

| 1. **Do you feel that your hypertension is under control?** | - Yes [1] - No [2] - Don’t Know [3] |
| --- | --- |
| 1. **Do you believe the medications are effective in reducing blood pressure levels?** | - Yes [1] - No [2] - Don’t Know [3] |

**SECTION 6: FACTORS INFLUENCING ADHERENCE**

| 1. **If you ever missed/stopped taking your medication(s) what was/were the reason(s)?** | - Side effects [1] - Forgetfulness [2] - Too many pills [3] - Cost of medication [4] - I believe I in divine intervention [5] - I believe(d) I am/was cured of hypertension [6] - I don’t believe orthodox medication can help the situation [7] |
| --- | --- |
| 1. **There are too many pills to be taken each day.**   **How many anti-hypertensive tablets do you take**  **daily?** | - - One [1]   - Two [2]   - Three [3]   - Four [4]   - Other____________________ |
